# Similarities and Differences in the Management of Patients with Osteoporotic Vertebral Fractures and Those with Rebound-Associated Vertebral Fractures Following Discontinuation of Denosumab

**DOI:** 10.3390/jcm12185874

**Published:** 2023-09-10

**Authors:** Athanasios D. Anastasilakis, Polyzois Makras, Julien Paccou, Ilias Bisbinas, Stergios A. Polyzos, Socrates E. Papapoulos

**Affiliations:** 1 Department of Endocrinology, 424 Military General Hospital, 564 29 Thessaloniki, Greece; 2 Department of Endocrinology and Diabetes, 251 Hellenic Air Force & VA General Hospital, 115 25 Athens, Greece; pmakras@gmail.com; 3 Department of Medical Research, 251 Hellenic Air Force & VA General Hospital, 115 25 Athens, Greece; s.e.papapoulos@lumc.nl; 4 Department of Rheumatology, University of Lille, 59000 Lille, France; julien.paccou@chu-lille.fr; 5 First Department of Orthopaedics, 424 Military General Hospital, 564 29 Thessaloniki, Greece; ibisbinas@hotmail.com; 6 First Laboratory of Pharmacology, School of Medicine, Aristotle University of Thessaloniki, 541 24 Thessaloniki, Greece; spolyzos@auth.gr; 7 Center for Bone Quality, Leiden University Medical Center, 2333 ZA Leiden, The Netherlands

**Keywords:** bone mineral density, denosumab, fracture, osteoporosis, rebound, vertebral

## Abstract

Rebound-associated vertebral fractures (RVFx) following denosumab discontinuation are typically multiple, are commonly associated with acute sharp pain, increase the risk of imminent fractures, and are pathogenetically different from common osteoporotic vertebral fractures (VFx). A clinically relevant question is whether patients with RVFx should be managed differently from patients with osteoporotic VFx. To address this question, we performed a systematic search of the PubMed database, and we reviewed current evidence on the optimal management of patients with RVFx. For pain relief of patients with RVFx, potent analgesics, often opioids, are essential. Information on the effectiveness of braces in these patients is scarce. Vertebroplasty and kyphoplasty are strongly contraindicated as they confer a substantial risk for new VFx. Exercise may be helpful, but again evidence is lacking. In contrast to patients with osteoporotic VFx, in whom initial treatment with bone-forming agents is recommended, patients with RVFx should initiate treatment with potent antiresorptives. To summarize, patients who have sustained RVFx following denosumab discontinuation are at a very high risk for new fractures, especially VFx. The management of such patients requires a multidisciplinary approach that should not be restricted to pain relief and administration of antiosteoporotic medication, but should also include back protection, early mobilization, and appropriate exercise.

## 1. Introduction

Denosumab (Dmab), a fully human monoclonal antibody that binds with high specificity to the receptor activator of nuclear factor κB ligand (RANKL), reduces osteoclast number and activity, thereby decreasing bone resorption [1]. Given to women with postmenopausal osteoporosis once every 6 months via SC injection, Dmab treatment was associated with a low incidence of fractures, low rates of adverse events, and continued increases in BMD without plateau up to 10 years [2]. Because of its efficacy and favorable safety profile, Dmab is widely used in the treatment of patients with osteoporosis.

In 2016, three different groups of investigators almost simultaneously described five women who stopped Dmab treatment and sustained multiple vertebral fractures (VFx) 8–16 months after the last injection [3,4,5]. A year later, a review of 24 cases (13 published and 11 new) supported the association of Dmab discontinuation with the occurrence of clinical and primarily multiple VFx [6]. A rapid increase in bone turnover markers above the pretreatment levels following the arrest of Dmab treatment [7] was thought to be related to the pathogenesis of these fractures, and the term “Rebound-associated Vertebral Fractures” (RVFx) was coined to differentiate them from osteoporotic VFx [3]. Similar to osteoporotic VFx, RVFx are usually localized in the lower thoracic and lumbar spine [6].

The crude incidence of all VFx following discontinuation of placebo or Dmab treatment, as recently estimated via a post hoc analysis of the FREEDOM and FREEDOM Extension studies, was 9.5% and 11.8%, while that of multiple VFx (≥2) was 3.7% and 7.2%, respectively [8]. Moreover, the proportion of multiple VFx was higher after stopping Dmab (61%) compared to the proportion observed after stopping placebo (about 39%). Notably, the risk of multiple VFx after denosumab discontinuation increased with increased duration of treatment [8].

The mechanisms of continuous bone gain upon Dmab treatment and of rapid bone loss following its cessation are still incompletely understood [9]. Upregulation of osteoclastogenesis and osteoclast activity had been, however, reported to critically contribute to the increased vertebral fragility following Dmab discontinuation. Compared to treatment-naïve women with osteoporotic VFx, those who had sustained VFx 8–16 months after the last injection of Dmab had significantly higher blood levels of RANK (13-fold) and cathepsin K (2.6-fold) mRNA and decreased levels of microRNAs involved in osteoclastogenesis downregulation (miR-503 and miR-222-2) [10]. In addition, patients with RVFx had higher numbers of TRAP-positive osteoclasts in their iliac crest bone biopsies than either treatment-naïve patients or patients on Dmab treatment [11]. Thus, although patients with RVFx and those with osteoporotic VFx share common risk factors, such as prevalent VFx, which present a significant risk factor for new fractures, these VFx differ pathophysiologically. A clinically relevant question is, therefore, whether the management of patients with RVFx should be different from that of patients with osteoporotic VFx. To address this question, we collected data reported during the past 7 years, and we summarize herein current views on the management of individuals with RVFx and how this might differ from the management of individuals with osteoporotic VFx.

## 2. Methods

### Literature Search

**Figure 1:**
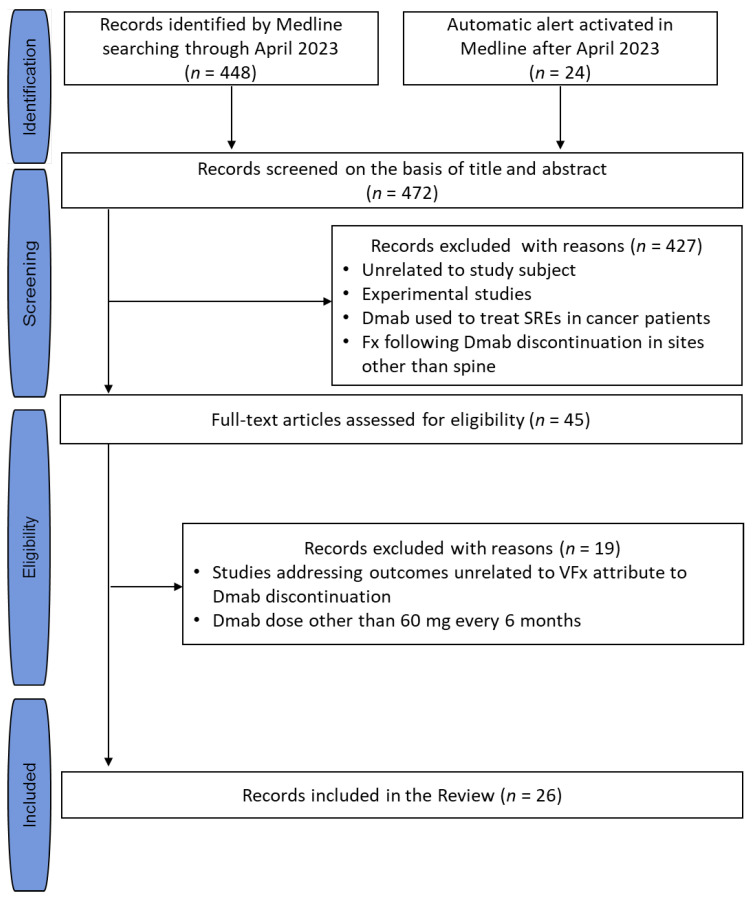
Flowchart of the search strategy and article selection process. Abbreviations: Dmab, denosumab; SREs, skeletal-related events; VFx, vertebral fracture.

An unrestricted computerized literature search was performed using the PubMed database. Based on the Medical Subject Heading (MeSH) terms, the following query was set: (“Spinal Fractures” [Mesh] OR (rebound-associated vertebral fractures) OR (vertebral fractures)) AND (“Denosumab” [Mesh] OR denosumab OR (receptor activator of nuclear factor kappa-Β ligand inhibitor)) AND (“Osteoporosis” [Mesh] OR osteoporosis)”. This search initially provided 448 articles (5 April 2023). Subsequently, an automatic alert was activated in PubMed (“My NCBI”) to retrieve relevant articles published after the initial search, which provided another 24 articles (last update 20 July 2023). The literature search was extended by “hand searching” the “related citations” linking to the selected relevant articles (first 20 articles per included article, after sorting according to relevance) and the references of all the selected relevant articles, which, however, retrieved no additional articles. The search was not limited by publication time or language. As indicated by the search items, articles reporting vertebral fractures following discontinuation of a Dmab dose other than 60 mg once every 6 months, as used in osteoporosis, were not considered. The article selection process is depicted in a flowchart (Figure 1).

## 3. Diagnosis

**Figure 2:**
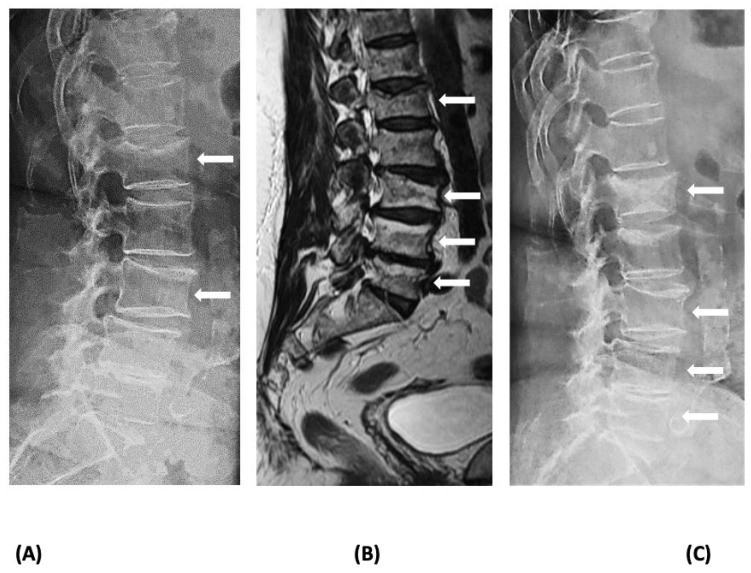
Imaging of a patient with rebound-associated vertebral fractures 10 months after the last denosumab dose (4 months off treatment): (**A**) lateral lumbar spine X-ray at the time of acute back pain onset showing fractures at L1 and L3; (**B**) magnetic resonance imaging of the spine at the same time reveals additional recent fractures at L4 and L5; and (**C**) lateral lumbar spine X-ray 3 months later depicting deterioration of the deformities of the vertebrae (collapse of the acute, non-collapsed vertebral fractures). Fractures are indicated with white arrows.

Vertebral compression fractures, which are the most common osteoporotic fractures, can cause significant back pain, limitation of activities, loss of independence, depression, chronic pain, and reduced quality of life, and increase the risk of imminent fractures and mortality [12,13]. While the most likely diagnosis in a patient presenting with a radiologically identifiable VFx 6 to 18 months after the last Dmab injection is RVFx, the recency of the fracture needs to be confirmed through an examination of earlier radiographs as back pain is a common symptom of elderly patients who may also have prevalent VFx [14,15,16]. If such radiographs are not available, recency can be verified using MRI, which can distinguish new from old VFx [17]. Severe back pain in a patient discontinuing or delaying Dmab treatment should always alert the physician of the possibility of RVFx, even if radiographs do not reveal spine deformities [17]. In such cases, described in the literature as acute non-collapsed VFx, a fracture may not be immediately identified but may be detected later in repeat radiographs; thus, MRI of the spine should be performed to confirm or exclude the diagnosis (Figure 2). In addition, other causes of VFx, e.g., malignancy, should be excluded. Establishing the diagnosis of RVFx may not be critical for the early management of patients, but it is essential for the longer-term therapeutic approach.

## 4. Management

Following the establishment of a correct diagnosis, the management of patients with VFx consists of short- and longer-term measures that include both non-pharmacological and pharmacological interventions. Short-term measures aim mainly at alleviating the symptoms, while long-term measures additionally aim at prevention of new vertebral fractures as well as non-vertebral osteoporotic fractures.

### 4.1. Pain Relief

#### 4.1.1. Pharmacological Interventions

Complaints of patients with osteoporotic VFx may vary considerably, ranging from lack of any pain (asymptomatic VFx) to sharp, breathtaking, and incapacitating pain [18]. In most cases, pain is tolerable and resolves without any treatment, and patients may recall a previous episode of pain when a VFx is incidentally identified on radiographs [19,20,21]. Acute pain usually subsides after 4–6 weeks but may persist for longer periods if the healing process is slower and can be replaced by chronic dull pain due to adjacent muscle spasms. Oral, nonopioid analgesics (acetaminophen, naproxen, and ibuprofen) are effective, especially in cases with mild to moderate pain [22]. Patients with severe pain will probably require oral or even parenteral opioids (tramadol, tapentadol, buplenorphine, hydrocodone, and oxycodone) [22]. Muscle relaxants may also reduce acute pain [23,24,25], although there is controversy regarding their ability to provide additional pain relief when they are combined with analgesics [26,27,28]. In contrast to osteoporotic VFx, RVFx are typically multiple and are commonly associated with acute [6], sharp pain that requires treatment with potent analgesics, often opioids [29]. In some cases, even opioids are ineffective in controlling the pain [30].

There are limited data about the use of antiosteoporotic agents in the management of acute pain of VFx. Intravenous pamidronate has been reported in controlled studies to provide greater pain relief compared to placebo [31] or calcitonin [32]. In a retrospective study, pain relief was achieved significantly earlier with denosumab than with alendronate [33]. Pain control was reported in two patients with RVFx treated with teriparatide [34] and denosumab [35], respectively. It should be emphasized that the use of teriparatide alone is contraindicated in patients who discontinue Dmab treatment [36] (see also Section 4.2).

#### 4.1.2. Non-Pharmacological Interventions

##### Bracing

In VFx, disproportionate height loss from the anterior vertebral body is common and results in wedging, which compromises the mechanical capacity of the spine to support daily activities and increases the loads on the anterior of the other vertebrae, thus predisposing patients to new VFx [37,38]. Conservative management with pharmacological and non-pharmacological interventions reduces symptoms and improves muscle flexibility and strength, but it does not decrease the anterior vertebral loading [38,39]. The latter can be reduced with bracing, which ensures a more upright posture that may prevent deterioration of the fracture configuration. Bracing could be used to relieve pain both in the acute and subacute phases of a VFx, but it is not routinely used and is not indicated in patients with mild symptoms [40]. Wearing a brace is quite inconvenient for patients, and it is generally not recommended for long periods due to the anticipated atrophy of the trunk muscles because of inactivity and the restricted respiration that reduces compliance [41]. At present, new dynamic semirigid orthoses are preferred over conventional, rigid spinal orthoses [41].

The use of orthoses in individuals with RVFx is rarely reported. The fact that RVFx are often multiple significantly increases the risk of worsening spine biomechanics and strength, resulting in more severe thoracolumbar kyphosis which, together with the weakening of the intact vertebrae due to high bone turnover, predisposes patients to additional VFx [37]. In this setting, bracing applied during the initial phase may theoretically reduce the loads in the vertebrae, at least until the rebound of bone turnover is controlled.

##### Vertebroplasty/Kyphoplasty

The majority of patients with symptomatic VFx are treated conservatively [42]. However, some patients may still experience excruciating or persistent pain. In such cases, treatment by vertebroplasty or kyphoplasty has been proposed, but studies on the effectiveness and safety of these interventions have provided variable and sometimes conflicting results [43,44,45,46]. Common and clinically important adverse events, such as occurrence of new VFx in the adjacent vertebrae in the months following the procedure [47,48,49], and cement extravasation [50,51] with sometimes life-threatening consequences [52,53], have decreased the use of these procedures in the management of patients with osteoporotic VFx.

In patients with RVFx, vertebroplasty and kyphoplasty are strongly contraindicated, as an increased risk of additional, most often multiple, fractures of the adjacent vertebrae has been described in several case series and case reports [6,54,55,56,57]. It is hypothesized that in patients discontinuing Dmab, even the intact vertebrae are seriously weakened due to the highly accelerated bone turnover and are susceptible to fractures [6]. Thus, when these intact vertebrae are subjected to increased compressing forces by the neighboring cemented vertebrae, they can easily fracture.

##### Exercise

There is no information about the effects of exercise on patients with RVFx, and measures recommended for patients with osteoporotic VFx are advisable. Notably, exercise regimens have been reported to decrease the use of analgesics and improve quality of life in some, but not all, studies [39,58]. Complete and long bedrest should be avoided, and patients should resume physical activity as quickly as possible [59]. In patients with the so-called vertebral fracture cascade, i.e., an increased risk of subsequent VFx after an initial VFx [60], weight-bearing and muscle-strengthening exercises, with caution to avoid undue stress on the back, are advised [37]. Hyperextension exercises may relieve pain, reduce subsequent VFx risk, and prevent kyphosis, while flexion exercises may increase subsequent VFx risk [61]. Posterior pelvic tilt exercise may also be helpful. Patients must be instructed to safely perform daily activities and avoid flexion of the spine, which, as mentioned above, increases loading on the anterior part of the vertebrae and has been associated with increased risk of new VFx [61]. A safe exercise program that includes back extension exercises, weight-bearing activities, and muscle-strengthening exercises to improve balance and upright posture is recommended.

### 4.2. Antiosteoporotic Medication

The presence of a VFx, even if this is asymptomatic, has significant clinical implications and substantially increases the risk of new fractures independently of BMD measurement [62]. This applies to prevalent VFx, but even more so to recent VFx, which confer a multifold increase in the risk of new fractures, especially in the two subsequent years [63], necessitating a more aggressive therapeutic approach. These notions, along with the fact that all efficacious antiosteoporotic medications perform better in treatment-naïve patients [8], have led to a shift in our approach to the optimal management of high-risk patients. It is currently recommended to initiate the treatment of patients at a very high risk of fractures with a bone-forming agent, such as stimulators of PTH1R (teriparatide and abaloparatide) or inhibitors of sclerostin (romosozumab), followed by a potent antiresorptive agent, such as bisphosphonates or denosumab [64]. Individuals who have sustained RVFx have a very high risk of fractures and are prone to imminent new fractures. Therefore, prompt treatment initiation is strongly recommended to prevent occurrence of additional VFx or worsening of already existing VFx [65]. However, despite the usually more severe presentation of RVFx compared to that of osteoporotic VFx, the choice of therapeutics does not follow the treatment advice for the latter. This is due to the different pathogenesis of RVFx, which requires initial treatment with an agent that rapidly and effectively reduces increased bone turnover. This should be given about 6 months after the last Dmab injection before the occurrence of RVFx, according to expert recommendation [36].

Cohort studies and prospective clinical trials have examined the efficacy of antiresorptives, mainly bisphosphonates, in preventing bone loss that follows Dmab discontinuation [66,67,68,69,70,71,72,73,74,75]. In most of these studies, intravenous zoledronate was used with different results depending on patient- and Dmab treatment-related factors [66,67,68,69,71,72], for example, prevalent VFx and duration of Dmab treatment, respectively [8,76]. Further discussion of these studies lies outside the scope of this review. Furthermore, it is uncertain whether the findings of these studies are applicable to the small group of patients who have already sustained RVFx and are at an even higher risk for new fractures. Cases with subsequent VFx after treatment of RVFx with Dmab, zoledronate, or teriparatide have been reported [77,78,79]. Thus, treatment should be preferably offered before the development of RVFx. In a large cohort study, Burckhardt et al. demonstrated a marked protective effect of bisphosphonates on the risk of RVFx in women who discontinued Dmab [80], a result supporting the current recommendations to treat all patients following discontinuation of Dmab. These findings together with the rarity of these events suggest that prospective studies should focus on optimal prevention of RVFx.

Regarding the use of bone-forming agents which, as mentioned above, are currently the preferred treatment of patients with severe osteoporosis, teriparatide should not be given alone as an initial therapy to patients with RVFx [36] as it may accelerate the already increased rate of bone turnover and reduce BMD at cortical sites [81]. A combination of teriparatide with bisphosphonate or with Dmab has not been tested in patients with RVFx. The inhibitor of sclerostin, romosozumab, is theoretically a more rational approach as it increases bone formation while decreasing bone resorption [82]. Given to women after discontinuation of Dmab, romosozumab was shown to increase BMD [83], but a case with RVFx following three injections of romosozumab given 9 months after the last Dmab injection was reported [84].

## 5. Conclusions

Patients with RVFx after Dmab discontinuation have a very high risk for new fractures, especially VFx. Data about the effectiveness of pharmacological treatments for such patients are limited, and there is almost no information about other modes of treatment or conservative management. The current recommendations to treat all patients discontinuing Dmab, coupled with the rarity of RVFx, make the planning of prospective, randomized clinical trials nearly impossible. Instead, efforts should be directed at elucidating the mechanism(s) of increased vertebral fragility and identifying the optimal strategy to prevent fractures. In addition, evaluation and identification of the most appropriate non-pharmaceutical interventions are necessary. Given the severity of the condition, we believe that a multidisciplinary approach for optimal management is warranted. Such an approach should not be restricted to pain relief and administration of antiosteoporotic agents, but should also include back protection, early mobilization, and appropriate exercise while excluding surgical interventions, such as vertebroplasty.

## Data Availability

Not applicable.
